# Exploring the Effectiveness of Technology-Assisted Interventions for Promoting Independence in Elderly Patients: A Systematic Review

**DOI:** 10.3390/healthcare12212105

**Published:** 2024-10-23

**Authors:** Mohammed Nasser Albarqi

**Affiliations:** College of Medicine, King Faisal University, Al Hofuf 31982, Saudi Arabia; aalbarqi@kfu.edu.sa

**Keywords:** elderly care, technology-assisted interventions, independence, telehealth, assistive technology

## Abstract

Background: The aging global population presents significant challenges for healthcare systems. Technology-assisted interventions have emerged as promising tools to enhance independence and well-being among elderly individuals. Objective: The aim of this study was to evaluate the effectiveness of technology-assisted interventions in promoting independence among elderly patients and identify key barriers and facilitators to their implementation. Methods: A systematic review was conducted following PRISMA guidelines. Searches were performed in PubMed, IEEE Xplore, ACM Digital Library, Cochrane Library, and Scopus. Studies evaluating technology-assisted interventions for promoting independence in elderly patients were included. Data were synthesized through narrative and thematic analysis. Results: Fourteen studies met inclusion criteria. Technology-assisted interventions demonstrated positive impacts on physical and cognitive functioning, health management, quality of life, and technological engagement among elderly patients. Improvements were observed in areas such as mobility, chronic disease management, mental health, and daily living activities. High usability and adherence rates were reported for well-designed interventions. However, challenges in user-centered design, personalization, and integration with existing healthcare systems were identified. Conclusions: Technology-assisted interventions show promise in promoting independence among elderly patients. Future research should focus on addressing identified challenges and conducting larger, long-term studies to confirm effectiveness and sustainability.

## 1. Introduction

The aging global population poses significant challenges and opportunities for healthcare systems worldwide [1,2]. As individuals age, there is a natural decline in physical and cognitive abilities, leading to increased dependency on healthcare services and caregivers [3]. Promoting independence among elderly patients is not only a quality of life issue but also crucial for reducing the burden on healthcare systems and supporting sustainable care practices [4]. In this context, technology-assisted interventions have emerged as promising tools to enhance the independence and overall well-being of elderly individuals [5].

Technology-assisted interventions encompass a wide range of tools and technologies designed to support the elderly in various aspects of their daily lives [6]. These interventions include telehealth services, wearable devices, smart home systems, and assistive robots, among others. Telehealth, for instance, has been shown to provide crucial healthcare services to elderly patients remotely, thereby reducing the need for frequent hospital visits and allowing older adults to maintain their independence for longer [7,8,9]. Studies have demonstrated that telehealth can effectively manage chronic diseases, a common issue in the elderly, through regular monitoring and timely intervention [10,11,12].

Wearable devices that monitor health parameters such as heart rate, blood pressure, and physical activity are also pivotal [13]. These devices empower elderly patients by providing them with real-time data about their health, enabling proactive management of their conditions [14]. Research indicates that wearables can significantly impact the management of chronic diseases by detecting potential health issues before they require acute intervention, thus maintaining an individual’s independence and reducing hospital admissions [15,16].

Smart homes equipped with sensors and Internet of Things (IoT) technology can provide a safe living environment for the elderly [17,18,19]. These technologies can automate tasks, remind patients to take medications, and alert caregivers in emergencies. Studies have shown that smart home technologies can greatly enhance the quality of life for the elderly by promoting safety and enabling them to perform everyday tasks more efficiently [20,21,22].

Assistive robots, another innovative technology, offer physical assistance and social interaction to elderly individuals. Robots can perform a range of functions from helping with day-to-day chores to providing companionship, addressing both the physical and emotional needs of the elderly [23]. Evidence suggests that robotic assistance can lead to improvements in mental health and social well-being, factors that are crucial for maintaining independence in later life [24].

Despite the potential benefits, the integration of technology in elder care is not without challenges. Issues such as technology acceptance, usability, and accessibility need to be addressed to ensure the effectiveness of these interventions [25]. Elderly individuals often face barriers to adopting new technologies due to physical limitations, cognitive impairments, or a lack of digital literacy. Therefore, designing age-appropriate, user-friendly technology solutions is critical for their successful adoption [26].

Furthermore, there are ethical considerations related to privacy, autonomy, and dependency that arise with the use of technology in elder care [27]. As technologies collect and analyze personal health data, ensuring the privacy and security of this information is paramount [28]. Moreover, while technology should aim to support independence, there is a delicate balance between providing necessary support and fostering over-reliance on technological aids [29].

As the global population continues to age, healthcare systems face increasing demands to support elderly individuals in maintaining their independence and quality of life. Technology-assisted interventions, such as telehealth, wearable devices, smart homes, and assistive robotics, have emerged as promising solutions to address these needs [30]. These technologies offer opportunities to monitor health, manage chronic diseases, improve mobility, and support daily activities, contributing to enhanced physical, cognitive, and emotional well-being in older adults [31].

In recent years, artificial intelligence (AI) has also emerged as a transformative force in healthcare, offering new possibilities for personalized and adaptive interventions. AI-driven systems have the potential to enhance existing technology-assisted interventions by providing real-time data analysis, predictive modeling, and personalized recommendations tailored to individual patient needs [32]. For example, AI can optimize telehealth platforms by predicting potential health risks based on patient data, allowing for earlier interventions and reducing hospitalizations. Additionally, AI-powered assistive robots can learn from user behavior and adapt their responses to better support individuals with cognitive impairments or mobility challenges [33].

### Aim of the Study

The aim of this systematic review is to evaluate the effectiveness of technology-assisted interventions in promoting independence among elderly patients. The study seeks to assess how various technologies, including telehealth, wearable devices, smart homes, and assistive robots, contribute to enhancing the autonomy and daily living activities of the elderly, while also considering the challenges and barriers to technology adoption within this demographic.

## 2. Materials and Methods

### 2.1. Search Strategy and Selection Criteria

This systematic review adheres strictly to the Preferred Reporting Items for Systematic Reviews and Meta-Analyses (PRISMA) guidelines to ensure comprehensive coverage and transparency in reporting. In line with the PRISMA statement, we crafted a detailed research protocol, which was duly registered with PROSPERO (CRD42024548885), underscoring our commitment to systematic rigor and methodological precision.

To thoroughly canvass the pertinent literature, extensive searches were performed across a broad array of prestigious databases, including PubMed, Cochrane Library, and Scopus. The search was conducted from database inception to September 10, 2024, aiming to include the most recent and relevant articles related to the scope of our investigation. Our search strategy was meticulously formulated, incorporating both medical subject headings (MeSH) and a carefully chosen array of keywords specifically relevant to the study of technology-assisted interventions for enhancing independence among elderly populations. Keywords and terms such as “telemedicine,” “e-health,” “mHealth,” “assistive technology,” “remote monitoring,” and “independence in elderly” were used to ensure all pertinent aspects of the subject matter were covered, including technological advancements, user-interface design tailored for the elderly, and evaluations of efficacy and user satisfaction of these technologies (Table 1).

### 2.2. Eligibility Screening

After the initial removal of duplicate entries, our systematic review process began with the screening of titles and abstracts, subsequently followed by an in-depth assessment of full-text articles. The inclusion criteria were designed to encompass original research articles, systematic reviews, meta-analyses, and clinical trials that involved human subjects. This review specifically targeted studies investigating technology-assisted interventions aimed at promoting independence among elderly patients. We focused on studies exploring various technological aids like telehealth systems, wearable devices, smart home technologies, and assistive robotics, and how these tools contribute to the autonomy and daily living of the elderly.

Included studies were those that evaluated the outcomes of these technological interventions on elderly independence, assessed the usability and acceptance of technologies by the elderly, or examined the impact of these technologies on health-related quality of life. Our primary outcomes of interest included improvements in functional independence, reductions in the need for caregiver support, enhanced patient safety, and overall satisfaction with the technology among elderly users.

Exclusion criteria were applied to non-research articles such as case reports, case series, opinion pieces, and editorials, as well as to studies conducted on animals or in vitro. Studies not specifically focusing on the elderly population or those involving interventions not aimed at promoting independence were excluded. Additionally, studies lacking rigorous methodological detail, those that did not provide comparative data or sufficient outcomes relevant to our research question, Finally, studies not available in English and without reliable translations were not considered.

### 2.3. Data Extraction

Data extraction was a pivotal phase in this systematic review, designed to gather and synthesize relevant information from selected studies concerning technology-assisted interventions aimed at promoting independence among elderly patients. The primary objective during this phase was to systematically compile critical data to illuminate the effectiveness, adoption challenges, and overall outcomes of these interventions in supporting elderly independence.

The data extraction process entailed a detailed review of each study, focusing on the following key components:

- Study Characteristics: We collected comprehensive information such as the study design, sample size, geographic location, publication date, and the demographic characteristics of the participants. This information is crucial for understanding the context of each study, assessing its relevance, and determining its applicability to this review.
- Technological Interventions: We extracted specific details about the technology-assisted interventions used in each study. This included the type of technology (e.g., wearable devices, telehealth platforms, smart home systems), its intended purpose, and the manner in which it was implemented to support elderly independence. Details about the implementation process, any technological adaptations made to suit the elderly population, and challenges encountered during implementation were also documented.
- Outcome Measures: We identified and documented the outcome measures employed by the studies to evaluate the effectiveness of the technological interventions. These measures included metrics such as improvements in the physical and cognitive independence of participants, user satisfaction, adoption rates, and any reported adverse effects or technological barriers.

In instances where data were missing, unclear, or incomplete, we reached out to the original authors of the studies for additional information or clarification, ensuring the accuracy and thoroughness of our data compilation. We also paid careful attention to potential overlaps in the study populations across the selected articles to avoid duplicity in our analysis. When uncertainties about patient cohorts arose, direct communication with the study authors was initiated to resolve these issues, maintaining the integrity and reliability of our data extraction process.

### 2.4. Quality Assessment

In this systematic review, the assessment of methodological quality and risk of bias in the included studies is paramount to ensure the credibility and applicability of the findings. This evaluation is critical, as it underpins our recommendations for the use of technology-assisted interventions to promote independence in the elderly.

For the quality assessment, we implemented a structured approach, using a modified version of the Cochrane Collaboration’s tool for assessing the risk of bias in randomized trials, alongside the ROBINS-I tool for non-randomized studies. These tools are renowned for their comprehensive evaluation frameworks, which are particularly suitable given the varied nature of interventions and settings in studies concerning elderly independence through technology.

Each study was independently reviewed to assess several key elements: study design, participant selection and categorization, fidelity to intervention protocols, methods of outcome measurement, and the handling of confounders and missing data. This detailed analysis is crucial for evaluating the internal and external validity of the studies and to identify any potential biases that could influence the results.

To maintain consistency and objectivity in our assessments, we addressed any discrepancies encountered during the review process with meticulous care and transparency. Differences in opinions regarding the risk of bias or methodological quality were resolved through a consensus-based approach. This involved in-depth discussions among the review team, ensuring that every decision regarding study evaluations was made collaboratively and unanimously.

### 2.5. Data Analysis

In our systematic review focusing on the effectiveness of technology-assisted interventions for promoting independence in elderly patients, data analysis was executed through a multi-faceted approach that combined both narrative synthesis and thematic analysis. This methodology enabled a comprehensive examination and integration of key findings from the selected studies, thereby enriching our understanding of how technological interventions can enhance elderly independence. Below is an outline of how each analytical method contributed to our overall analysis:

1. Narrative Synthesis: Serving as the foundation of our data analysis, narrative synthesis allowed for an in-depth review of the collected data, going beyond mere aggregation of results. This approach facilitated a critical examination of the literature, enabling us to synthesize information from a variety of studies. Our focus was on evaluating the practical implementation, effectiveness, and user reception of various technological interventions such as wearable devices, telehealth systems, smart home technologies, and robotic assistants. The narrative synthesis provided a detailed, contextualized overview of the studies, highlighting key trends, emerging challenges, and opportunities within the domain of technology-assisted interventions aimed at improving the autonomy of elderly individuals. This synthesis not only clarified the effectiveness of these technologies but also illuminated the conditions under which they were most beneficial.
2. Thematic Analysis: The thematic analysis followed a structured approach to identify key themes within the selected studies. We conducted the analysis in three main steps:
  1. Data Familiarization: Initially, all data relevant to the objectives were extracted from each study, focusing on aspects such as user adaptability, technological accessibility, patient safety, and comfort. This step involved reviewing each study’s findings in depth to ensure a comprehensive understanding.
  2. Coding Process: We systematically coded data by identifying and labeling distinct units of meaning across the studies. Codes were generated inductively based on observed patterns within the studies. Two researchers independently coded the data, and any discrepancies were discussed and resolved to ensure consistency.
  3. Theme Identification and Refinement: Using the initial codes, we identified recurring themes across the studies, particularly those that highlighted facilitators and barriers in technology adoption for elderly patients. Themes were then refined by grouping similar codes and prioritizing those frequently mentioned or critical to independence outcomes (e.g., adherence to technology, ease of use). A consensus was reached on final themes after thorough discussion among the research team.

### 2.6. Quality Assessment and Validity Criteria

The internal and external validity of the studies included in this systematic review were assessed using well-established frameworks. Internal validity refers to the degree to which the study accurately measures the intervention’s effects without bias or confounding factors, while external validity refers to the extent to which the results can be generalized to other populations or settings.

To ensure a rigorous evaluation of both internal and external validity, the following criteria were applied:

1. Internal Validity:
  ∘ Randomization and Allocation Concealment: We assessed whether studies employed appropriate randomization methods to reduce selection bias. For non-randomized studies, we evaluated the use of other strategies such as matching or controlling for confounding variables. Allocation concealment was examined to determine whether the allocation sequence was adequately hidden from participants and investigators to prevent selection bias.
  ∘ Blinding: We evaluated the use of blinding for participants, personnel, and outcome assessors. Studies were categorized as high or low risk depending on whether blinding was adequately implemented. Lack of blinding in some domains, particularly for outcome assessors, was considered a potential source of detection bias.
  ∘ Measurement of Outcomes: The use of validated and reliable instruments for measuring outcomes was evaluated. We assessed whether the studies clearly defined primary and secondary outcomes and whether these measures were consistently applied throughout the study.
  ∘ Handling of Missing Data: We reviewed how studies managed missing data and whether appropriate methods such as intention-to-treat analysis were used to minimize bias. Studies that failed to report on missing data or used inappropriate handling methods were considered to have a higher risk of bias.
2. External Validity:
  ∘ Population Representativeness: The demographic characteristics of the study populations were analyzed to determine how representative they were of the broader elderly population. Studies conducted on highly specific or restricted populations (e.g., limited to a single geographic area or patients with a specific condition) were noted as having limited generalizability.
  ∘ Intervention Applicability: We examined whether the interventions used in the studies could be feasibly applied in real-world settings. Factors considered included the complexity of the intervention, the level of technical support required, and the availability of resources (e.g., telehealth infrastructure).
  ∘ Follow-Up and Long-Term Impact: We assessed the follow-up periods in each study to determine whether the long-term effects of the intervention were evaluated. Studies with short follow-up periods were considered to have limited external validity as they might not capture the sustainability of the intervention’s effects.

## 3. Results

### 3.1. Included Studies

**Figure 1:**
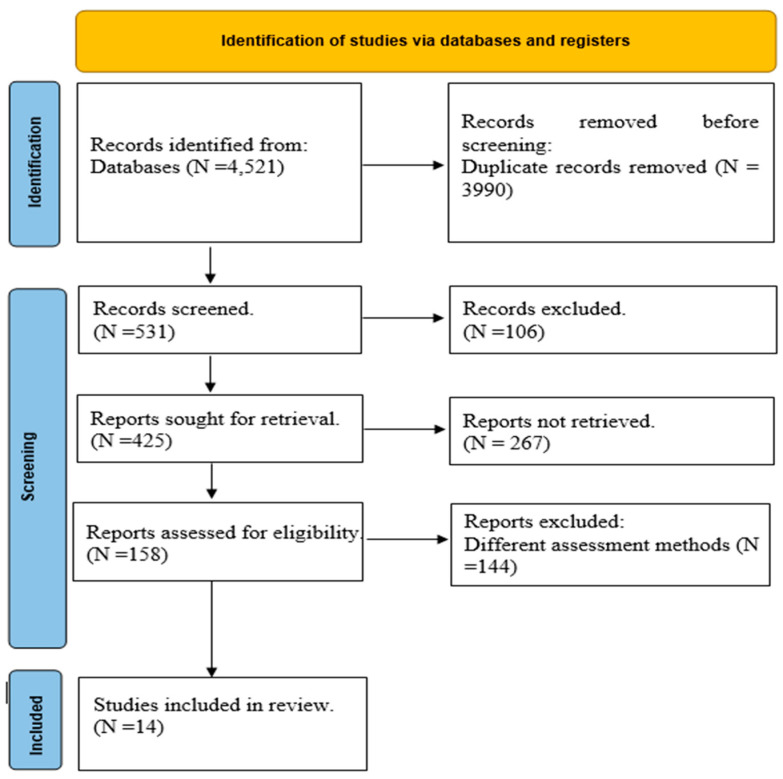
PRISMA flowchart of the included studies.

From the initial 4521 documents identified, duplicates were removed, leaving 531articles for preliminary screening based on titles and abstracts. Of these, 106 articles were excluded at this stage, leaving 425 papers for further detailed eligibility assessment. After a comprehensive review of the full texts, 14 studies met all inclusion criteria and were selected for inclusion in this systematic review. The entire selection process, along with reasons for exclusion at various stages, is meticulously documented in a PRISMA flowchart (Figure 1), which will be included in the final report to ensure transparency and reproducibility of the review process.

### 3.2. Risk of Bias Assessment

**Figure 2:**
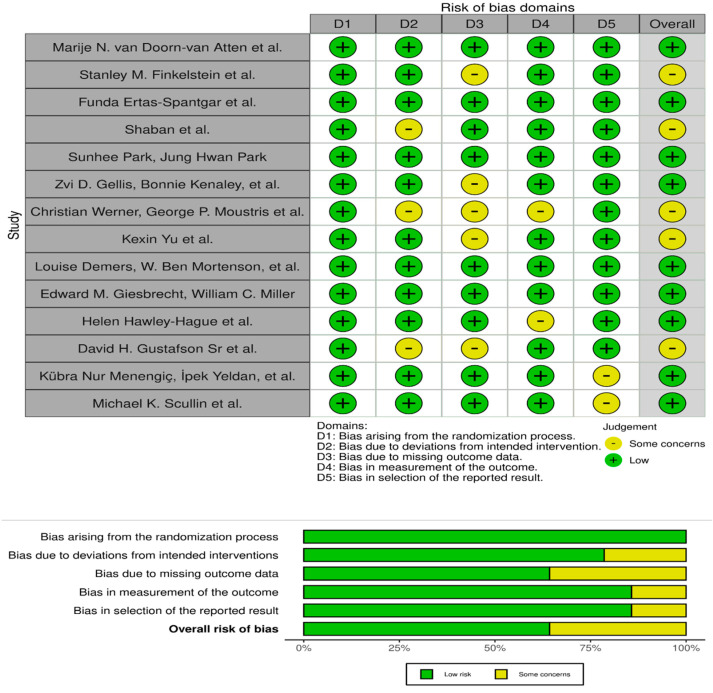
Risk of bias assessment [34,35,36,37,38,39,40,41,42,43,44,45,46,47].

The risk of bias assessment for the studies included (Figure 2) in this review generally indicates a low risk across most evaluated domains, underscoring the methodological rigor employed in the majority of the research [34,35,36,37,38,39,40,41,42,43,44,45,46,47]. Notably, several studies, such as those by Marije N. van Doorn-van Atten et al. and Funda Ertas-Spantgar et al., maintained a consistent low-risk profile across all domains, likely reflecting strong random sequence generation, proper allocation concealment, and effective blinding of outcome assessments. However, certain studies like those by Stanley M. Finkelstein et al. and Shaban et al. demonstrated some concerns, particularly in domains related to the blinding of participants and personnel, which could introduce performance and detection biases. More pronounced concerns were evident in studies by Christian Werner, George P. Moustris et al., and David H. Gustafson Sr et al., where multiple domains raised issues, especially around blinding and allocation, indicating potential impacts on the study’s findings due to possible biases in participant behavior and outcome reporting. This varied risk profile across studies highlights the complex nature of clinical and intervention research, particularly when blinding participants and personnel is challenging or impractical. It also underscores the importance of employing rigorous methodologies and the necessity for careful interpretation of results, particularly in studies where methodological weaknesses are identified. Overall, the presence of some concerns in specific domains suggests a need for ongoing scrutiny and methodological enhancement in future research to ensure the reliability and applicability of findings in the field of technology-assisted interventions for the elderly.

### 3.3. Main Outcomes

The data extracted from various studies [34,35,36,37,38,39,40,41,42,43,44,45,46,47] on technology-assisted interventions for elderly care have been synthesized into four distinct thematic outcomes, illustrating the diverse impacts and potential benefits of integrating technology into elderly healthcare and daily living support (Table 2).

A key finding across the studies was the importance of user-centered design and adequate training in the successful adoption of technology-assisted interventions. Thematic analysis revealed that interventions tailored to the individual’s cognitive and physical abilities, with ongoing technical support, were more likely to be adopted and sustained over time. This suggests that future technology development should prioritize personalization and usability, ensuring that older adults can effectively engage with the technology.

#### 3.3.1. Physical and Cognitive Functioning

One of the prominent themes focuses on improvements in both physical and cognitive functioning. Research such as the studies by Kübra Nur Menengiç et al. and Edward M. Giesbrecht and William C. Miller demonstrates how motor-cognitive dual-task exercises via telerehabilitation and mHealth applications for wheelchair skills can significantly enhance mobility, cognitive functions, and daily living tasks [39,46]. These interventions are particularly beneficial for elderly individuals with impairments or chronic conditions, suggesting that tailored exercise programs and cognitive interventions can effectively support the physical and mental health of this demographic.

#### 3.3.2. Health Management and Disease Control

Another critical theme is health management and disease control, where technological interventions play a crucial role in managing chronic diseases such as diabetes and heart conditions. Studies like those conducted by Shaban et al. and Zvi D. Gellis and Bonnie Kenaley show that digital tools can facilitate better disease management through improved monitoring, personalized care, and timely interventions [38,44]. These interventions lead to improved HbA1c levels, enhanced self-care activities, and reduced hospital visits, ultimately enhancing patient autonomy and reducing healthcare costs.

### 3.4. Quality of Life and Mental Health

The impact of technology on quality of life and mental health is also significant. Interventions such as the ElderTree platform studied by David H. Gustafson Sr et al. and smartphone apps for fall prevention by Helen Hawley-Hague et al. have been shown to improve mental quality of life, increase social support, and reduce the incidence of fall [47]. These improvements not only aid in maintaining independence but also help mitigate the effects of isolation and depression among the elderly, enhancing their overall well-being and social interactions.

#### Technological Usability and Adherence

Lastly, the theme of technological usability and adherence highlights the importance of the design and implementation of user-friendly technological tools. High usability ratings, good adherence to intervention protocols, and positive participant engagement with technology, as noted in studies by Michael K. Scullin et al. and again by Edward M. Giesbrecht and William C. Miller, indicate that the elderly are capable of effectively engaging with technology when it is designed to meet their specific needs and capabilities [34,39].

These thematic outcomes collectively underscore the varied and significant benefits of technology-assisted interventions in elderly care. They emphasize the need for interventions that are not only medically beneficial but also accessible and engaging for elderly users, suggesting a multidimensional approach to integrating technology into elderly healthcare practices. This integration has the potential to transform the quality of life and independence of elderly individuals, having a profound impact on their ability to live fulfilling lives with reduced healthcare burdens.

## 4. Discussion

This systematic review has examined the effectiveness of technology-assisted interventions in promoting independence among elderly patients. The findings reveal a complex landscape where various technological solutions show promise in enhancing the autonomy, health management, and overall quality of life for older adults. However, the implementation and adoption of these technologies are not without challenges.

### 4.1. Effectiveness of Technology-Assisted Interventions

The reviewed studies demonstrate that technology-assisted interventions can significantly impact physical and cognitive functioning, health management, quality of life, and technological engagement among the elderly. These findings align with the growing body of evidence supporting the integration of technology in elderly care [48,49].

One of the most promising outcomes observed across multiple studies is the improvement in both physical and cognitive functioning. The study by Kübra Nur Menengiç et al. [46] showed significant improvements in cognitive and mobility functions, as well as functional independence, among early–middle-stage Alzheimer’s disease patients through a telerehabilitation program. Similarly, Edward M. Giesbrecht and William C. Miller [39] reported improved wheelchair skills and self-efficacy among older adults using manual wheelchairs through an mHealth application. These findings suggest that tailored technological interventions can effectively support physical and cognitive health in the elderly, even those with existing impairments or chronic conditions [50].

The potential of technology to enhance physical functioning is further supported by Helen Hawley-Hague et al. [47], whose study on smartphone apps for fall prevention showed promising results in terms of adherence and potential improvements in balance and function. This aligns with previous research highlighting the importance of fall prevention in maintaining independence among the elderly [51].

Health Management and Disease Control Several studies in this review demonstrated the efficacy of technology-assisted interventions in managing chronic diseases, a critical aspect of maintaining independence in older adults. Shaban et al. [44] and Sunhee Park and Jung Hwan Park [45] both reported significant improvements in diabetes management through digital interventions. These findings are particularly noteworthy given the prevalence of chronic diseases in the elderly population and their impact on independence [52].

The study by Zvi D. Gellis and Bonnie Kenaley [38] on telehealth interventions for older adults with heart failure or COPD showed improvements in health and social functioning, along with reduced emergency department visits. This aligns with previous research on the potential of telehealth to reduce healthcare utilization and costs while improving patient outcomes [53,54,55,56].

The impact of technology on quality of life and mental health emerged as a significant theme. David H. Gustafson Sr et al. [40] found that their eHealth intervention, ElderTree, led to better outcomes in mental quality of life and social support among high primary care users. This underscores the potential of technology to address not just physical health but also the psychosocial needs of the elderly, which are crucial for maintaining independence and well-being [57,58].

The study by Kübra Nur Menengiç et al. [46] also reported reductions in anxiety and depressive symptoms through their telerehabilitation program, highlighting the potential of technology to support mental health in the elderly population. This is particularly important given the high prevalence of mental health issues among older adults and their impact on overall functioning and independence [59].

A critical factor in the success of technology-assisted interventions is their usability and the ability of elderly users to adhere to their use. Michael K. Scullin et al. [34] reported high usability and adherence rates for smartphone apps designed to support prospective memory in older adults with mild cognitive impairment or mild dementia. This finding is encouraging as it suggests that even elderly individuals with cognitive impairments can effectively engage with well-designed technological interventions [34].

Similarly, the study by Edward M. Giesbrecht and William C. Miller [39] on an mHealth application for wheelchair skills training showed good engagement and significant improvements in self-efficacy. These findings challenge the notion that older adults are unable or unwilling to engage with new technologies and support the potential for widespread adoption of technology-assisted interventions in this population [60].

### 4.2. Challenges and Considerations

While the reviewed studies generally show positive outcomes, several challenges and considerations emerged that warrant further attention in the development and implementation of technology-assisted interventions for the elderly.

The success of interventions like those reported by Michael K. Scullin et al. [34] and Edward M. Giesbrecht and William C. Miller [39] underscores the importance of user-centered design in creating technologies for the elderly. Future interventions should prioritize ease of use, clear interfaces, and adaptability to varying levels of technological literacy to ensure widespread adoption and effectiveness [61].

The varied nature of the elderly population, with differing health conditions, cognitive abilities, and technological experience, highlights the need for personalized and adaptable interventions. Future research should explore how technology-assisted interventions can be tailored to individual needs and preferences to maximize their effectiveness and adoption [62,63].

While many of the reviewed studies showed promising results, the challenge of integrating these technologies into existing healthcare systems remains. Future research should focus on how technology-assisted interventions can be seamlessly incorporated into current care practices, ensuring continuity of care and maximizing their potential to support independence [64].

Many of the reviewed studies were of relatively short duration, ranging from a few weeks to several months. There is a need for longer-term studies to assess the sustained effectiveness of these interventions and their impact on long-term independence and health outcomes in the elderly population [65].

As technology becomes more integrated into elderly care, ethical considerations around privacy, data security, and autonomy become increasingly important [66,67]. Future research and implementation efforts should address these ethical concerns to ensure that technology-assisted interventions enhance rather than compromise the dignity and independence of elderly individuals.

The heterogeneity of the interventions examined, including telehealth systems, wearable devices, and assistive robots, reflects the broad scope of technology-assisted solutions for elderly care. While this diversity presents challenges in direct comparison, it allows for the identification of overarching trends and factors that influence the success of these technologies. Rather than comparing individual interventions, this study synthesizes common facilitators and barriers, such as ease of use, personalization, and integration with healthcare systems, that are crucial for promoting elderly independence.

Factors such as the patient’s educational level, the presence of comorbidities, and their ability to communicate effectively with the device significantly influenced the outcomes of the interventions. Studies that accounted for these variables reported better adherence and outcomes, underscoring the need for future research to control for such factors when evaluating the efficacy of technology-assisted interventions for the elderly.

### 4.3. The Role of Artificial Intelligence (AI)

Artificial intelligence (AI) holds considerable promise for enhancing the effectiveness of technology-assisted interventions for elderly care. AI has the potential to address some of the barriers identified in this review, particularly around personalization and usability [22].

AI-powered systems can be used to create more personalized interventions by analyzing individual user data and adapting the technology to their specific needs and preferences [68]. For instance, AI can predict when an elderly individual may need assistance based on patterns in their behavior, allowing for more proactive and timely interventions. Furthermore, AI can improve the user experience by enabling more intuitive and adaptive interfaces, particularly for elderly users who may struggle with conventional technology [69].

AI can also assist in overcoming accessibility barriers by offering real-time support through voice-activated systems and automated learning tools that help users navigate technology with minimal difficulty [70]. In terms of healthcare system integration, AI can facilitate better coordination between technological interventions and clinical care, ensuring that healthcare providers are informed in real-time about the health status of elderly patients. This could lead to more comprehensive and integrated care for elderly individuals who are using technology-assisted interventions [71].

### 4.4. Limitations and Future Directions

This review has several limitations that should be considered. First, the heterogeneity of the interventions and outcome measures across studies made direct comparisons challenging. Future research could benefit from more standardized outcome measures to facilitate meta-analyses and more direct comparisons between interventions.

Second, many of the included studies had relatively small sample sizes and short durations, limiting the generalizability of their findings. Larger, long-term studies are needed to confirm the effectiveness and sustainability of technology-assisted interventions in promoting independence among the elderly.

Third, while this review focused on the effectiveness of interventions, it did not extensively explore the cost-effectiveness of these technologies. Future research should include economic evaluations to assess the feasibility of widespread implementation of technology-assisted interventions in elderly care.

Lastly, most of the reviewed studies were conducted in developed countries with relatively high levels of technological infrastructure. There is a need for research on the applicability and effectiveness of these interventions in diverse global contexts, including low- and middle-income countries.

## 5. Conclusions

This systematic review set out to evaluate the effectiveness of technology-assisted interventions in promoting independence among elderly patients. The findings from the included studies demonstrate that telehealth, wearable devices, smart home technologies, and assistive robots all contribute to improving autonomy in various areas, including mobility, chronic disease management, mental health, and daily living activities. These interventions show great potential in maintaining and enhancing the quality of life for elderly individuals.

However, several challenges remain, including difficulties with usability, lack of personalization, and the need for better integration with existing healthcare systems. Addressing these barriers is critical for ensuring the broader adoption and sustained use of these technologies in elderly care. Future research and development should focus on optimizing these interventions to meet the unique needs of elderly populations, ensuring that they are accessible, user-friendly, and integrated seamlessly into healthcare workflows.

In conclusion, technology-assisted interventions show promise in promoting independence among elderly patients, but their success depends on addressing key barriers and ensuring that these technologies are designed with the end user in mind.

## Figures and Tables

**Table 1:** Search Strategy.

Database	Search Terms
PubMed	(“Elderly”[Mesh] OR “Aged”[Mesh] OR “Older adults”) AND (“Technology-Assisted Interventions”[Mesh] OR “Telehealth” OR “Wearable Devices” OR “Smart Home” OR “Assistive Technology”) AND (“Independence”[Mesh] OR “Autonomy” OR “Self Care”[Mesh])
MEDLINE	Same as PubMed
Embase	(‘elderly’/exp OR ‘aged’/exp OR ‘older adults’) AND (‘technology-assisted interventions’/exp OR ‘telehealth’ OR ‘wearable devices’ OR ‘smart home’ OR ‘assistive technology’) AND (‘independence’/exp OR ‘autonomy’ OR ‘self care’/exp)
Web of Science	TS = ((elderly OR aged OR “older adults”) AND (“technology-assisted interventions” OR telehealth OR “wearable devices” OR “smart home” OR “assistive technology”) AND (independence OR autonomy OR “self care”))
Cochrane Library	(“Elderly” OR “Aged” OR “Older adults”) AND (“Technology-Assisted Interventions” OR “Telehealth” OR “Wearable Devices” OR “Smart Home” OR “Assistive Technology”) AND (“Independence” OR “Autonomy” OR “Self Care”)
Google Scholar	(“Elderly” OR “Aged” OR “Older adults”) AND (“Technology-Assisted Interventions” OR “Telehealth” OR “Wearable Devices” OR “Smart Home” OR “Assistive Technology”) AND (“Independence” OR “Autonomy” OR “Self Care”)
Scopus	(TITLE-ABS-KEY (elderly OR aged OR “older adults”) AND TITLE-ABS-KEY (“technology-assisted interventions” OR telehealth OR “wearable devices” OR “smart home” OR “assistive technology”) AND TITLE-ABS-KEY (independence OR autonomy OR “self care”))

**Table 2:** The extraction table.

Authors	Country	Study Design	Sample Size	Population Characteristics	Type of Technology	Intervention Details	Duration of Intervention	Control Group	Outcomes Measured	Main Findings
Marije N. van Doorn-van Atten et al. 2018 [35]	The Netherlands	Non-randomized controlled design	214	Average age 80, community-dwelling older adults	Telemonitoring	Multi-component intervention: self-measurements of nutritional outcomes and physical activity, education, follow-up by a nurse	6 months	Regular care	Diet quality, physical activity, fruit intake, protein intake, saturated fatty acids intake	Intervention increased self-monitoring and knowledge, improved perceived behavioral control for physical activity. Mediated effects on diet quality and intake of protein.
Stanley M. Finkelstein et al. 2006 [37]	USA	Randomized Controlled Trial	80	Elderly, average age 80.3, managing chronic conditions	Telehealth	Telehealth platform including broadband internet, videoconferencing, a web portal for services, and physiological monitoring; focus on the usage of the ordering service portal.	6 to 9 months	Traditional care	Usage patterns of telehealth services, independence in self-care, user interaction with technology.	Effective use of telehealth platform, improvement in maintaining independence and self-care capabilities.
Funda Ertas-Spantgar et al. 2024 [36]	Germany	Randomized Controlled Trial	24	Stroke patients with severe dressing impairment, including those with neglect and/or apraxia	RehaGoal App, Errorless Learning Techniques	Errorless learning (EL) with backward chaining and method of vanishing cues, using the RehaGoal App for training dressing tasks	2 weeks	Standard therapy in the rehab unit	Nottingham Stroke Dressing Assessment, Barthel Index, Functional Independence Measure	No significant improvement in dressing ability with the intervention. Neglect and apraxia were predictors of non-improvement.
Shaban et al. 2024 [44]	Egypt	Quasi-experimental	120	Adults with type 2 diabetes, aged 18+, from outpatient clinics	Digital mobile application	Digital-based nursing intervention using a mobile app providing personalized education on diabetes management	4 months	Standard care (routine visits and printed materials)	Knowledge of diabetes management, self-efficacy, and self-care activities (diet, exercise, medication adherence, glucose testing, foot care)	Significant improvement in knowledge, self-efficacy, and self-care activities in the intervention group compared to control
Sunhee Park, Jung Hwan Park, 2024 [45]	South Korea	Randomized Controlled Trial	120	Older adults with type 2 diabetes, average age ~73	Mobile app (DiaNote)	Digital self-care intervention using the DiaNote app for diabetes management, including educational sessions, self-recording, monitoring, and nurse-led phone consultations	12 weeks	Traditional logbook	HbA1c levels, diabetes self-care activities, self-efficacy, quality of life	The intervention led to improved HbA1c control and was as effective as traditional logbooks for enhancing quality of life and self-care activities.
Zvi D. Gellis, Bonnie Kenaley et al. 2012 [38]	USA	Randomized Controlled Trial	102	Homebound older adults with HF or COPD	Telehealth	Telehealth intervention with in-home monitoring, educational and care management support by a telehealth nurse, integrated with electronic medical records	12 months	Usual care + education	Health-related quality of life, mental health, service utilization, satisfaction with care	Improved health and social functioning, decreased depression symptoms, and reduced emergency department visits in the intervention group compared to control.
Christian Werner, George P. Moustris et al. 2017 [42]	Germany, Greece	Randomized Controlled Trial	42	Frail older adults with and without cognitive impairment using rollators	Robotic Rollator (RR)	RR provided navigation assistance with audio cues to assist in navigation through a hospital setting.	Single session	No navigation assistance (participants used conventional signposts for navigation)	RR-assisted navigation improved navigation performance, especially in participants with cognitive impairment, reducing completion and stopping times significantly.	Small sample size and short duration limited generalizability. The study did not report any severe limitations or adverse events during the testing.
Kexin Yu et al. 2020 [43]	Taiwan	Randomized Controlled Trial	97	Older adults with Type 2 Diabetes Mellitus (T2DM), average age 65+	mHealth App (IMTOP app)	Intergenerational Mobile Technology Opportunities Program (IMTOP): 8-week technology and diabetes self-management training followed by 4-week technical support, facilitated by college students	8 months	Usual care	Self-care behaviors, T2DM symptoms, clinical outcomes, health resource utilization, medical expenditure	Significant improvements in diet, exercise, smoking, and blood glucose testing at 4 months. Reduced clinic visits and medication costs. Increased reporting of diabetes symptoms possibly due to heightened awareness.
Louise Demers, W. Ben Mortenson et al., 2016 [41]	Canada	Randomized Controlled Trial	120 dyads	Older adults (>55 years) with mobility limitations and their caregivers	Assistive Technology	Home-based, tailored AT intervention focusing on the needs of both older adults and their caregivers, including caregiver training	1 year	Customary care	Functional autonomy, caregiver burden, quality of life, health service utilization	N/A
Edward M. Giesbrecht, William C. Miller, 2019 [39]	Canada	Feasibility Randomized Controlled Trial	18	Older adults using manual wheelchairs, able to self-propel	mHealth	mHealth application for wheelchair skills training; included 2 in-person sessions and 4 weeks of home practice with a tablet focusing on wheelchair skills	6 weeks	Tablet games focusing on cognitive and dexterity training	Improved wheelchair skills, self-efficacy, and participation; significant effects in participation and self-efficacy, with medium to large effect sizes	Small sample size, short intervention duration
Helen Hawley-Hague et al., 2023 [47]	UK	Feasibility Randomized Controlled Trial	50	Community-dwelling older adults at risk of falls, aged 50+	Smartphone Apps	“Motivate Me” and “My Activity Programme” apps supporting falls rehabilitation with exercises, feedback, and goal setting	6 months	Standard care with basic app functionality for recording exercise	Feasibility of the intervention, recruitment rates, adherence, dropout rates, balance, function, falls, strength, fear of falling, health-related quality of life, resource use	Feasible intervention with positive indications from outcome measures; higher adherence in the intervention group; no significant adverse events related to the apps
David H. Gustafson Sr et al., 2022 [40]	USA	Randomized Clinical Trial	390	Older adults, ≥65 years, with health challenges	eHealth (ElderTree)	Access to ElderTree, an interactive website designed to improve quality of life, social connection, and independence	12 months	Usual access to information and communication	Quality of life, independence, social support, depression, falls prevention	No main effects of ElderTree over time, except better outcomes in mental quality of life and social support among high primary care users
Kübra Nur Menengiç, İpek Yeldan et al., 2022 [46]	Turkey	Online Pilot Randomized Controlled Trial	20	Early–middle-stage Alzheimer’s disease patients	Telerehabilitation via Video Conferencing	Motor-cognitive dual-task exercises; 6-week program with real-time video conferencing sessions. Included physical and cognitive tasks to improve both mobility and cognitive functions	6 weeks	No intervention	Cognitive functions, mobility, activities of daily living, functional independence, anxiety, depression, caregiver’s well-being	Significant improvements in cognitive and mobility functions, functional independence, and reduction in anxiety and depressive symptoms.
Michael K. Scullin et al., 2022 [34]	USA	Randomized Controlled Trial	52	Older adults, 74.79 ± 7.20 years, diagnosed with MCI or mild dementia	Smartphone Apps	Two groups: one using a reminder app and the other a digital voice recorder app to support prospective memory. Training provided for both groups.	4 weeks	Not specified	Prospective memory performance, daily functioning, quality of life, and usability of technology	Significant improvements in prospective memory and daily functioning, high usability and adherence to technology use.

## Data Availability

Data are available upon request.
